# Optical determination of crystal phase in semiconductor nanocrystals

**DOI:** 10.1038/ncomms14849

**Published:** 2017-05-17

**Authors:** Sung Jun Lim, André Schleife, Andrew M. Smith

**Affiliations:** 1 Department of Bioengineering, University of Illinois at Urbana-Champaign, 1270 Digital Computer Laboratory MC-278, Urbana, Illinois 61801, USA; 2 Micro and Nanotechnology Laboratory, University of Illinois at Urbana-Champaign, 208 North Wright Street MC-249, Urbana, Illinois 61801, USA; 3 Intelligent Devices and Systems Research Group, DGIST, 333 Techno Jungang-Daero, Hyeonpung, Daegu 42988, Republic of Korea; 4 Department of Materials Science and Engineering, University of Illinois at Urbana-Champaign, Materials Science and Engineering Building MC-201, 1304 West Green Street, Urbana, Illinois 61801, USA

## Abstract

Optical, electronic and structural properties of nanocrystals fundamentally derive from crystal phase. This is especially important for polymorphic II–VI, III–V and I-III-VI_2_ semiconductor materials such as cadmium selenide, which exist as two stable phases, cubic and hexagonal, each with distinct properties. However, standard crystallographic characterization through diffraction yields ambiguous phase signatures when nanocrystals are small or polytypic. Moreover, diffraction methods are low-throughput, incompatible with solution samples and require large sample quantities. Here we report the identification of unambiguous optical signatures of cubic and hexagonal phases in II–VI nanocrystals using absorption spectroscopy and first-principles electronic-structure theory. High-energy spectral features allow rapid identification of phase, even in small nanocrystals (∼2 nm), and may help predict polytypic nanocrystals from differential phase contributions. These theoretical and experimental insights provide simple and accurate optical crystallographic analysis for liquid-dispersed nanomaterials, to improve the precision of nanocrystal engineering and improve our understanding of nanocrystal reactions.

## Abstract (web summary)

Identifying crystallographic phases in solution is not possible with standard diffraction methods. Here, Lim *et al*. demonstrate the *in situ* identification of cubic and hexagonal phases of cadmium selenide nanocrystals using optical methods based on first-principles electronic theory.

Semiconductor nanocrystals (NCs) are light-absorbing, light-emitting materials with diverse applications that span bioimaging^1^,^2^,^3^,^4^, light-emitting devices^5^,^6^,^7^, solar cells^8^,^9^,^10^ and consumer electronics^11^. These nanoparticles exhibit unique optical and electronic properties that derive from the crystalline nature of electronic bonding, tunable by composition, size, shape and crystal phase^12^. In recent years, solution-based syntheses have generated diverse and complex NC structures incorporating multiple chemical and crystalline domains within single particles, providing new and useful emergent properties^13^,^14^,^15^,^16^. To predict and understand the electronic and optical properties of these complex materials, a critical initial step is structural analysis using materials science techniques such as electron microscopy for morphology and diffractometry for crystal structure. However, these low-throughput methods are a significant bottleneck in the synthesis pipeline that limits the capacity to explore synthesis conditions in high-throughput and to monitor NC growth in real-time. Therefore, a major advance was the discovery that NC size and concentration quantitatively correlate with features of steady-state absorption spectra that can be measured rapidly in solution^17^,^18^,^19^,^20^,^21^,^22^,^23^.

Unfortunately, crystal phase remains challenging to measure in high-throughput. Powder X-ray diffraction (XRD) and electron diffraction require pure materials fixed on a solid substrate and are not compatible with NCs dispersed in solution. This hinders analysis for colloidal samples and may also introduce structural changes due to purification-mediated removal of surface adatoms and ligands needed to prevent aggregation or fusion^24^,^25^,^26^,^27^. Moreover, diffraction patterns become less clear as the NC size decreases, because reduced atomic periodicity broadens scattering peaks^28^,^29^. This is especially important for NCs composed of II–VI compounds (for example, CdS, CdSe), III–V compounds (InAs, GaP) and I–III–VI_2_ compounds (CuInS_2_, CuInSe_2_) that exist in two distinct crystal structures, zinc blende (ZB) and wurtzite (WZ), which can both be present in the same NC (polytypism)^30^,^31^. Diffraction patterns of ZB and WZ phases only subtly differ and can be nearly indistinguishable when NCs are very small or polytypic^28^,^30^.

Distinguishing ZB and WZ is critically important, because phase is a key design parameter for tuning NC structure and optical properties. Phase-specific synthesis methods now allow growth of size-tunable NCs with either ZB or WZ phases for CdS^32^,^33^, CdSe^33^,^34^ and CdTe^35^ (CdE) compositions. A major outcome is that NC shape is determined by phase, as the high symmetry of ZB tends to yield spheres, tetrahedrons or cubes, whereas the WZ phase tends to yield rod and disk-shaped NCs^13^,^36^,^37^,^38^,^39^. The two phases can be combined to yield structures like tetrapods with ZB cores from which four WZ rods extend outward in a tetrahedral geometry and different chemical components can be incorporated in the different domains^33^,^40^,^41^. Phase also has an important impact on the types and spatial arrangement of crystalline facets on the NC surface, as ZB facets tend to be isotropic, whereas the hexagonal structure of WZ requires a multi-faceted surface with different electrostatic charges, anisotropic reactivity and an intrinsic electrostatic polarization^42^,^43^. The anisotropic WZ structure can also exhibit anisotropic optical properties and unique interactions with polarized light^44^,^45^.

In this study, we introduce a new methodology for NC crystallography. We demonstrate that polymorphic ZB and WZ NCs can be reliably distinguished based on optical signatures measured through steady-state absorption spectroscopy from samples dispersed in liquids. Focusing on CdSe and using a combination of experiment and parameter-free electronic-structure theory, we demonstrate that distinct absorption features (*E*_1_ transitions) at energies much higher than band-edge *E*_0_ transitions clearly distinguish WZ from ZB crystal structures. This allows high-throughput optical assessment of crystal phase from *E*_1_ transitions, independently from *E*_0_ features that provide size and concentration information. Therefore phase characterization can be performed in liquid suspension and the high sensitivity of absorption spectroscopy allows the use of small quantities of material. This technique also provides unambiguous signatures for NCs with very small sizes (∼2 nm) for which XRD patterns provide vague phase information. We also show evidence that *E*_1_ transition features enable a semi-quantitative classification of polytypic structures by differential contributions from WZ and ZB phase-specific transitions. However, substantial nonlinearity is observed with polytypism, an effect that derives from differences in electronic band sharing between different phase domains. We further demonstrate that this procedure allows monitoring of crystal phase during dynamic NC processes. Finally, we describe the photophysical origins of *E*_1_ and *E*_0_ transitions, and discuss the applicability of these findings to other polymorphic NC materials.

## Results

### Phase-dependent absorption spectra of CdSe NCs

**Figure 1:**
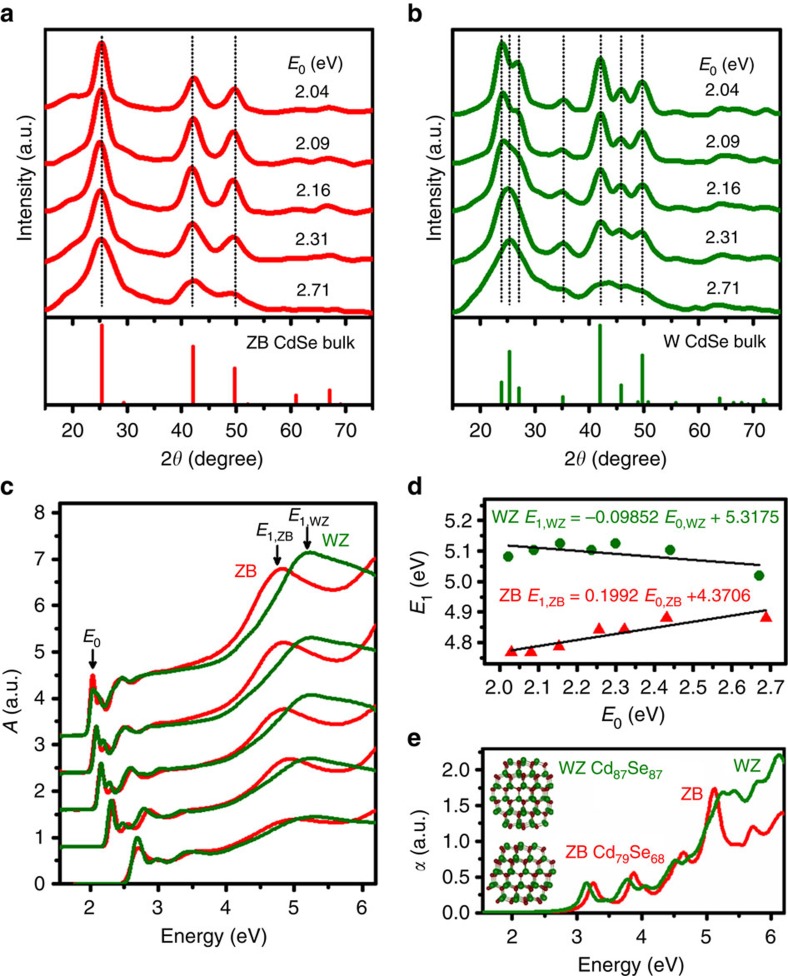
Phase-dependent absorption spectra of CdSe NCs. (**a**,**b**) XRD patterns of CdSe NCs with five different *E*_0_ transitions, matched between ZB (**a**) and WZ (**b**). Bulk patterns are shown below the experimental spectra with vertical dotted lines corresponding to the major diffraction peaks. (**c**) Experimental absorption spectra of ZB and WZ CdSe NCs, with matched *E*_0_ transitions. Prominent crystal phase-specific *E*_1_ bands are apparent at ∼4.9 eV for ZB and ∼5.1 eV for WZ. (**d**) Measured relationship between *E*_0_ and *E*_1_ for ZB (red triangles) and WZ (green circles) CdSe NCs with linear fits (black lines), showing little quantum confinement effects for *E*_1_ in comparison with *E*_0_ and a greater size-dependent shift of *E*_1_ for ZB compared with WZ. (**e**) DFT-simulated absorption coefficient (*α*) spectra of ZB (red) and WZ (green) CdSe NCs, showing similar *E*_0_ transitions and phase-distinct *E*_1_ bands in the 5–6 eV range. The structures used in the simulations are depicted in the plot inset.

We synthesized a size series of CdSe NCs using high-temperature organic-phase procedures that yield quasi-spherical particles. ZB NCs were synthesized from cadmium carboxylates and selenium dioxide at 220–230 °C, and WZ NCs were prepared from cadmium phosphonates and trioctylphosphine selenide at 300–365 °C (detailed methods are in Supplementary Notes 1 and 2)^33^,^34^. We prepared and rigorously purified monodisperse sets of WZ and ZB NCs with matched band-edge transitions, which we call *E*_0_ peaks, synonymous with the ‘first exciton' peaks. As shown in Fig. 1a,b, distinctive signatures of WZ and ZB crystal phases were evident from powder XRD by comparison with bulk standards, although distinctive features were not apparent for the smallest NCs with *E*_0_ near 2.7 eV (∼2.0 nm NC diameter). Figure 1c shows ultraviolet-visible absorption spectra of these NCs dispersed in hexane in the 1.55–6.20 eV region (200–800 nm). Well-known features of quantum confinement are apparent for both ZB and WZ, including a shift of *E*_0_ transitions to higher energies with decreasing NC size, discrete transitions near the band-edge and a featureless, monotonic rise in absorbance with increasing energy^12^. Interestingly, band-edge spectral regions are similar when comparing ZB and WZ particles^46^, but large deviations are present in high-energy spectral regions between 4 and 6 eV. We designate peaks in this spectral region as *E*_1_ in convention with their bulk origins (see below)^47^ and demonstrate that their energies uniquely correlate with crystal phase: *E*_1,ZB_ is ∼4.9 eV and *E*_1,WZ_ is ∼5.1 eV. It is important to note that these peaks are not readily apparent when spectra are plotted in wavelength scale due to compression of spectral features at short wavelengths (Supplementary Fig. 1)^48^.

Figure 1d depicts the relationship between *E*_0_ and *E*_1_ peaks for CdSe NCs, extracted through the second derivative of each spectrum (Supplementary Fig. 2)^49^. The two phases are highly distinguishable by *E*_1_ peak energy for all NCs, even for small NCs with *E*_0_ transitions near 2.7 eV (460 nm), for which broad scattering bands in XRD spectra wash out distinguishing features (Fig. 1a,b and Supplementary Fig. 3). *E*_1_ peak energies have little dependence on NC size compared with *E*_0_ transitions, which we evaluate in detail below. Figure 1e shows CdSe NC spectra simulated using density functional theory (DFT) that clearly show transitions in the 5.0–5.5 eV range that are similar to those in experimental spectra, with the ZB peak at lower energy than the WZ peak. For these simulations, we used a semi-local Perdew–Burke–Ernzerhof exchange-correlation functional^50^,^51^ for which quasiparticle energies were approximated using a scissors operator (also used for bulk CdSe below) and optical transition-matrix elements were computed within the longitudinal approximation^52^ using the Vienna *Ab-initio* Simulation Package^53^,^54^. These quasi-spherical WZ or ZB CdSe NCs were composed of 147–174 atoms and passivated by pseudo-hydrogen atoms to saturate dangling bonds. The NCs are smaller than those tested experimentally, due to the large computational cost of DFT for large numbers of atoms. Although this causes the computed spectra to be blue-shifted compared with experiments, the relative trend between WZ and ZB is reliable. We also note that excitonic effects are not included due to large computational costs of many-body perturbation theory for NCs. However, we verify (see below) that excitonic effects are small for bulk CdSe and do not substantially impact the trends our conclusions are based on.

### Relation to band-edge features and ligands

**Figure 2:**
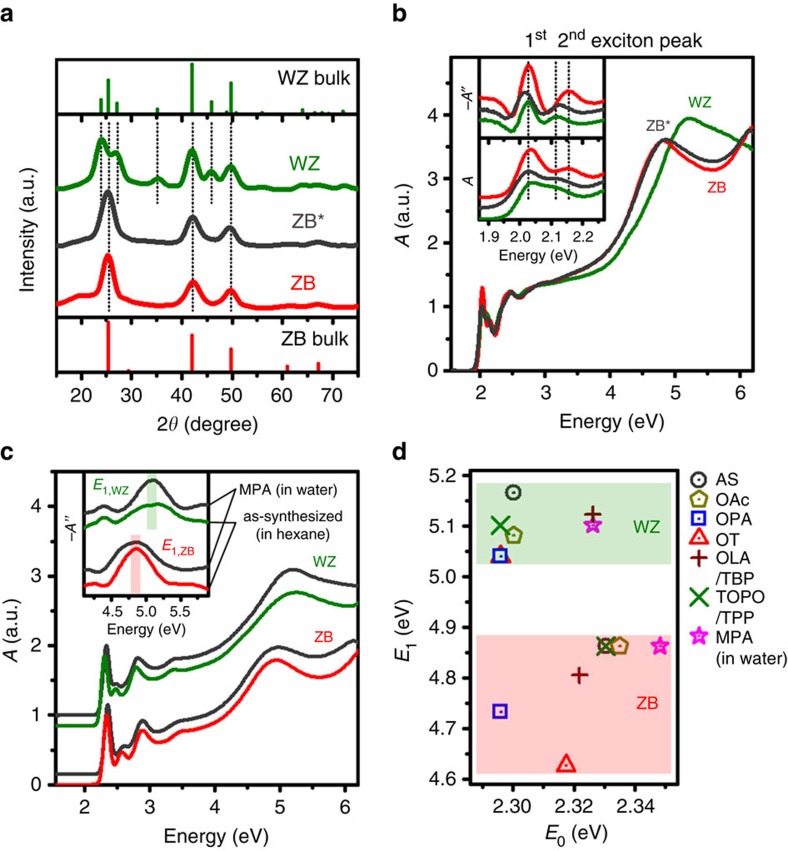
Relationships between absorption features and ligands. (**a**) Powder XRD patterns of three CdSe NCs with standard ZB (red) and WZ (green) phases, as well as a ZB CdSe nanocrystal synthesized in the presence of sodium oleate (ZB*, grey). (**b**) Absorption spectra of the same three CdSe NCs, comparing the *E*_0_ and *E*_1_ peaks. The *E*_1_ peaks accurately reflect the underlying crystal structure, but the band-edge transitions of ZB* look the same as an *E*_0_-matched WZ nanocrystal rather than ZB. The inset shows the zeroth and second derivative of the absorption spectra band-edge regions, showing distinct differences between the standard ZB and WZ spectra, but the ZB* spectrum exhibits a band-edge almost identical to standard WZ. (**c**) Absorption spectra of as-synthesized ZB and WZ NCs with matched band-edge energies in hexane, in comparison with their spectra after ligand exchange with MPA in aqueous solution. Insets show second derivatives of the spectra to depict their distinguishable phase-dependent *E*_1_ transitions. Red and green shades indicate the energy ranges of *E*_1,ZB_ and *E*_1,WZ_ peaks, respectively, which are clearly separated. (**d**) Extracted *E*_0_ and *E*_1_ transitions for WZ and ZB NCs as-synthesized (AS) and after ligand exchange with the indicated ligands. OAc, oleic acid; OLA, oleylamine; OPA, octylphosphonic acid; OT, octanethiol; TBP, tributylphosphine; TOPO, trioctylphosphine oxide; TPP, triphenylphosphine.

Previous reports suggested that CdSe NCs exhibit absorption features near the band edge that distinguish different phases^55^,^56^. We compared many sets of NCs with matched *E*_0_ transitions and observed that *E*_1_ absorption transitions were a much more reliable indicator of phase than any band-edge feature. It was previously observed that the difference in energy between the two lowest energy absorption transitions, the first and second exciton peaks, is smaller for WZ NCs compared with ZB NCs^56^; however, no theoretical model or systematic empirical data has substantiated this correlation. On the contrary, we find that band-edge transitions are highly sensitive to surface effects and ligands. For example, as shown in Fig. 2, we found surface conditions that reverse the previously observed band-edge energy trend for ZB and WZ CdSe NCs. Figure 2a shows XRD patterns of WZ and ZB NCs grown using standard methods described above, in addition to a third NC that clearly demonstrates a ZB crystal structure, denoted as ZB*. These NCs were synthesized using the typical synthetic protocol for ZB NCs, but with the addition of sodium oleate, which we experimentally observed shifted the second exciton peak, but had little other impact on the NC structure or absorption spectrum shape. Figure 2b shows that *E*_1_ absorption features for ZB* remained accurately indicative of the ZB phase; however, the *E*_0_ absorption transitions looked similar to those of WZ in terms of the first and second exciton peaks (see second derivatives of spectra in inset). Clearly, *E*_1_ transitions are more accurate probes for phase than band-edge transitions, which are sensitive to changes in surface structure, shape and adatoms^25^,^49^,^57^,^58^,^59^,^60^.

We further analysed the impact of ligands on *E*_1_ transition features. We exchanged native hydrophobic ligands on as-synthesized ZB and WZ NCs with hydrophilic thiols (mercaptopropionic acid, MPA). As shown in Fig. 2c, *E*_1_ transition features were conserved after this process, even in a solvent with a 42-fold larger dielectric constant than hexane. We also evaluated the impact of hydrophobic ligands with diverse chemistries by measuring *E*_0_ and *E*_1_ energies for ZB and WZ NCs in hexane after ligand exchange (Supplementary Note 3). We only evaluated ligands which colloidally stabilized the NCs and simultaneously prevented oxidative etching (Supplementary Fig. 4). We compared oleic acid (OAc), octylphosphonic acid, octanethiol (OT), oleylamine (OLA) with tributylphosphine (TBP) and trioctylphosphine oxide (TOPO) with triphenylphosphine. Fourier-transform infrared spectroscopy confirmed that MPA and OT completely replaced ligands on both NC phases, and phosphonates completely replaced carboxylates on ZB NCs. Exchange was less efficient in other cases, yielding mixed ligand coatings (Supplementary Figs 5 and 6). Importantly, three different pairs of NCs with ZB or WZ phases had equivalent ligands (MPA, OT and phosphonates) and, in all cases, *E*_1_ remained in its phase-specific spectral window. As shown in Fig. 2d, some ligands induced little shift (<100 meV) of *E*_1_ (OAc, MPA, OLA/TBP and TOPO/triphenylphosphine), but OT and octylphosphonic acid yielded substantial red shifts for both *E*_0_ and *E*_1_, especially for ZB NCs. Interestingly, *E*_1_ shifts were not always proportional to *E*_0_ shifts, which may be due to how the electronic energy levels of ligands align with NC electronic energy bands.

We further note that ligands and solvents had only minor contributions to extinction near *E*_1_ peaks. It was necessary to acquire spectra with proper background subtraction to eliminate solvent impacts, and hexane and water were found to be optimal due to high transparency in the critical range of 4.6–5.2 eV (240–270 nm). Although their impact could not be equivalently subtracted, the primary ligands used had molar extinction coefficients less than 400 M^−1^ cm^−1^ in this range, which is much smaller than the 10^6^–10^7^ M^−1^ cm^−1^ values for NCs (Supplementary Fig. 7). Assuming that approximately 100 ligands bind to ∼2.3 nm NCs and approximately 350 bind to ∼4.2 nm NCs, ligand absorption should contribute <3% of the total absorption. Importantly, spectra can be substantially skewed if NCs are not properly purified from their reaction solutions, which contain high concentrations of ligands. Purification was simple, but methods must be validated for specific ligand classes (Supplementary Fig. 8). For example, complete removal of phosphonate ligands required liquid–liquid extraction at 60 °C. Although we used multi-step procedures for purification to exhaustively eliminate free ligands in this work, a single non-solvent precipitation was normally sufficient for accurate *E*_1_-based phase identification.

### Polytypic NCs

**Figure 3:**
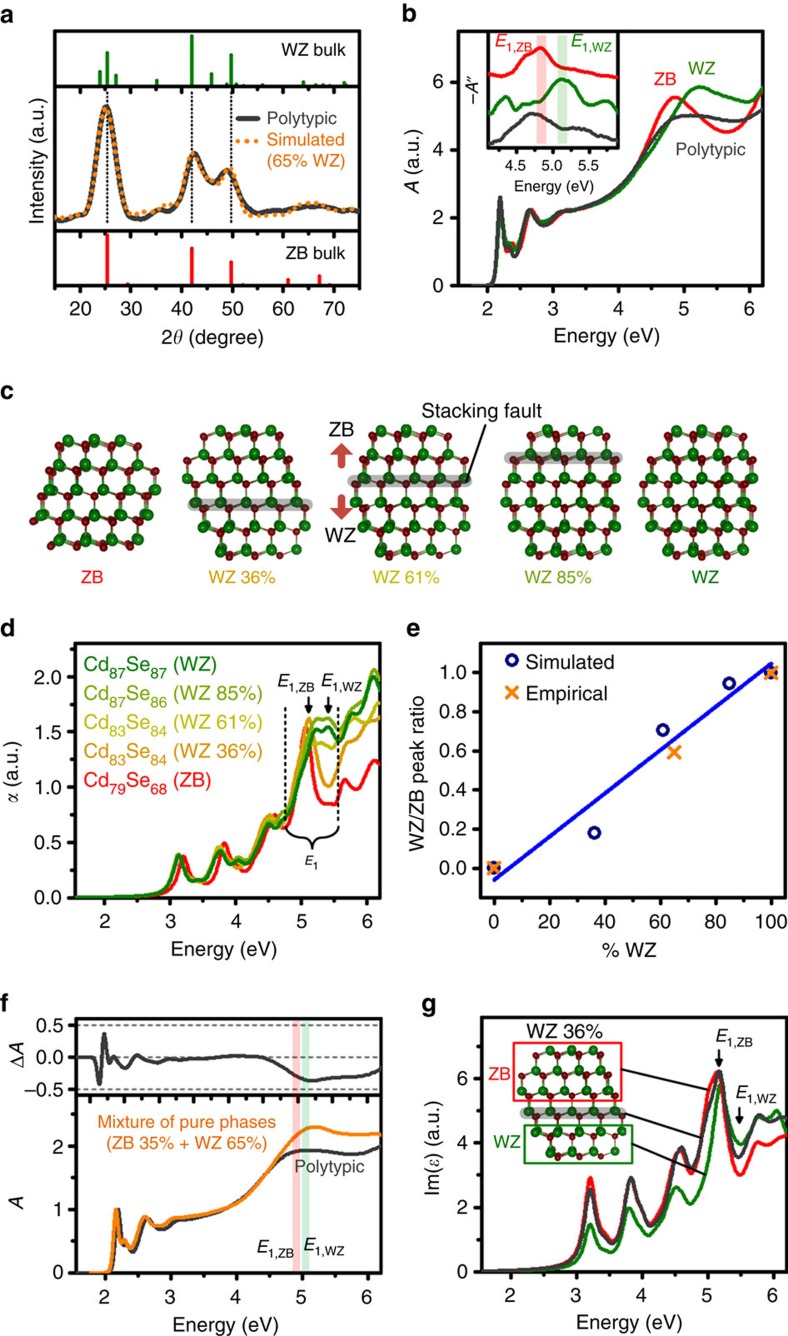
Spectral trends of polytypic CdSe NCs. (**a**) Powder XRD pattern of a polytypic CdSe NC with ∼3.6 nm diameter (solid grey line) and simulated pattern with 65% WZ and 35% ZB (orange dotted line). The major diffraction peaks for the bulk ZB and WZ materials are also shown, with vertical dotted lines correspond to the shared features. (**b**) Absorption spectra of WZ (green), ZB (red) and polytypic (grey) NCs with matched *E*_0_ energies, showing that the polytypic *E*_1_ region is intermediate between those of WZ and ZB NCs. The inset shows the second derivative spectra, with red and green shades indicating the energy ranges of *E*_1,ZB_ and *E*_1,WZ_ peaks. (**c**) Structures of CdSe NCs with pure ZB or WZ phases or different degrees of polytypism (0, 36, 61, 85, 100% WZ) used in DFT simulations. ZB and WZ domains are separated by stacking faults in grey shading. (**d**) Simulated absorption spectra of nanocrystals shown in **c**. (**e**) Trend in the relative intensities of the two transitions, *A*_WZ_/*A*_ZB_, normalized to 0 for pure ZB and 1 for pure WZ, showing simulated data from **c** (blue circles) with a linear fit (blue line) and empirical data from **b** (orange crosses). (**f**) Spectrum of polytypic NCs (grey) in comparison with a summed spectrum from pure-phase ZB and WZ NCs in the correct mixture of phases (orange), showing that the experimental polytypic nanocrystal exhibits a depleted *E*_1,WZ_ peak. Red and green shades indicate the energy ranges of *E*_1,ZB_ and *E*_1,WZ_ peaks, respectively. (**g**) Theoretical spectra (imaginary component of the dielectric function) of the 36% WZ polytypic NC, broken down by domain region. All valence band states were used, but the conduction band was segregated into the indicated domains. In all domains, the ZB peak is evident, but the WZ peak has been substantially diminished.

We further evaluated spectral features of polytypic CdSe NCs with mixed WZ and ZB domains separated by stacking faults, synthesized by established reaction conditions using primary amine ligands^61^. Figure 3a shows an example XRD pattern for a 3.6 nm polytypic NC, showing a structure intermediate between phase-pure ZB and WZ patterns (see Fig. 1a,b). To quantitatively determine the underlying phase contributions to this polytypic sample, we used the Debye equation to simulate 1,024 XRD patterns of ∼3.6 nm NCs with different polytypic structures (see Methods for details)^28^. We then fit the empirical pattern with these simulated basis patterns using the least squares method to calculate the average polytypism from the averaged contribution of each structure to the final fitting. This yielded an average 65% contribution from WZ phase and 35% contribution from ZB phase. Figure 3b shows the optical spectrum of this polytypic NC together with phase-pure, *E*_0_-matched ZB and WZ NCs. As would be expected, the general shape of the *E*_1_ peak is intermediate between that of WZ and ZB, with a flat feature spanning the range of *E*_1_ energies of both WZ and ZB. This suggests that each WZ and ZB crystal domain individually contributes to the high-energy absorption features. However, the polytypic spectrum is clearly not a linear combination of the two phase-pure spectra, with lower total oscillator strength in this spectral region compared with the pure phase spectra.

To further evaluate *E*_1_ transitions in polytypic NCs, we relied on simulations due to the inability to precisely synthesize polytypic structures spanning a wide range of phase contributions. We simulated the spectra of three fully relaxed WZ–ZB polytypic NCs using DFT and compared them with phase-pure ZB and WZ spectra. Figure 3c shows the structures used, with stacking faults between ZB and WZ phases. Figure 3d shows that all spectra were similar below 5 eV, but showed clear phase-dependent trends in higher energy *E*_1_ transitions. However, the experimental observation of a total decrease in oscillator strength in the *E*_1_ transition region was not corroborated in the simulated results, probably due to the small sizes of these simulated NCs (see discussion below). Nevertheless, the theoretical results did correlate with the experimental results when plotted as the ratio of absorption intensity for the ZB phase near 4.9 eV, *A*_ZB_, to that of the WZ phase near 5.1 eV, *A*_WZ_. Figure 3e shows that the pure-phase normalized trend of *A*_WZ_/*A*_ZB_ increases for the theoretical spectra with increasing WZ phase fraction, and that the experimentally observed value falls along the trend. The deviation from a perfect linear trend for the theoretical data derives from the differing numbers of atoms and the differing Cd:Se stoichiometry across the simulated polytypic NCs that result from the need to maximize surface atom coordination numbers to prevent unrealistic intra-bandgap electronic states and weakly bound states.

We compared the experimental polytypic CdSe spectrum with a spectrum of phase-pure ZB and WZ NCs, mixed at the underlying polytype ratio (Fig. 3f). By subtracting the spectra, it is clear that the *E*_1,WZ_ transition is selectively depleted compared with the *E*_1,ZB_ transition. We evaluated this effect using theoretical polytypic spectra, subdivided by the phase domain from which the conduction band orbitals derive (Fig. 3g). The ZB domain and the interfacial domain exhibited spectral shapes resembling that of pure-phase ZB. However, in the WZ domain, the *E*_1,WZ_ peak was substantially depleted, which is consistent with experimental spectra. The origin of this effect is described below. Despite the nonlinearity in the individual absolute peak absorption values, this nevertheless allows a ratiometric analysis between *E*_1_ peaks to analyse polytypism. Unfortunately, validated synthetic methods for polytypic NCs spanning a wide range of phase compositions have not yet been reported and extensive DFT analyses are computationally limiting; thus, comprehensive corroboration of these findings for polytypic materials is still ongoing. It is further important to note that experimental spectra of polytypic NCs derive from heterogeneous populations of NCs in which stacking fault number and location may be randomly or non-randomly distributed, which can complicate correlations with simulations of individual NCs. However, our simulated NCs were prepared to resemble what we believe are prevalent materials in the distribution.

### Monitoring dynamic structural changes

**Figure 4:**
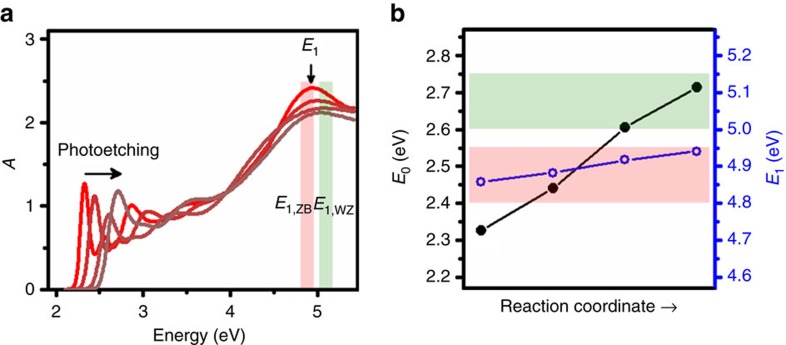
Process monitoring with *E*_1_ transition assessment of phase. (**a**) Absorption spectra of ZB CdSe NCs subject to photochemical etching, normalized by intensity at 4 eV. Curves are colour-coded to indicate the continuous shift in *E*_0_ to higher energy, with little shift in *E*_1_. (**b**) Trends in *E*_0_ (black solid circles) and *E*_1_ (blue open circles) transitions as the NC size decreases. Energy regions for *E*_1,ZB_ and *E*_1,WZ_ are indicated by red and green shading, respectively. Lines connecting data points are guides to the eye.

We demonstrate the capacity to easily monitor NC phase during chemical reactions with this optical methodology. We measured spectral changes arising from photochemical etching of ZB CdSe NCs that reduce the NC size and blue-shift the *E*_0_ peak. Figure 4a shows NC spectra (normalized at 4 eV), measured by removing aliquots during the etching process. Figure 4b shows the trends in *E*_0_ and *E*_1_ over the etching reaction coordinate. *E*_0_ increased by ∼0.4 eV as the NCs etched from 3 to 2 nm. In comparison, *E*_1_ exhibited little change (< 0.1 eV) indicating the absence of a phase change, as would be expected from a reaction mechanism that degrades the NC from the outside^62^.

### Structural origin of phase-dependent absorption features

**Figure 5:**
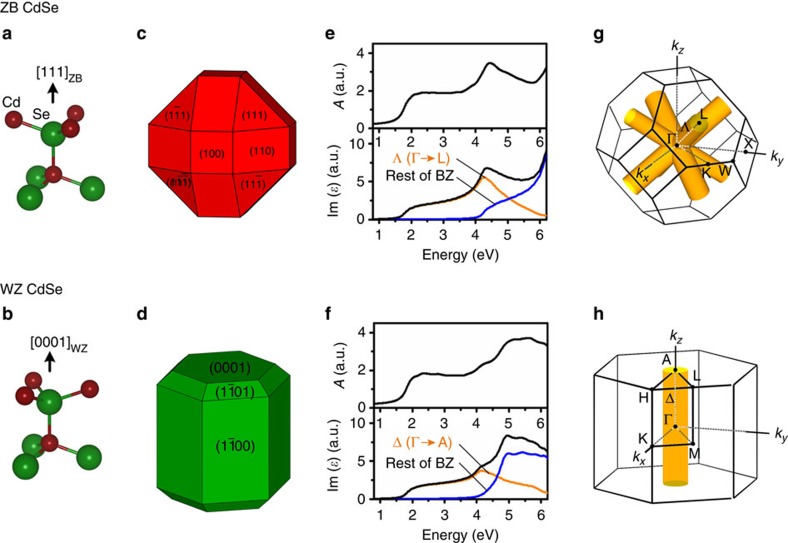
Structures and calculated absorption spectra of bulk CdSe in ZB and WZ crystal phases. Bonding in (**a**) ZB and (**b**) WZ crystal phases, showing staggered (ZB) and eclipsed (WZ) conformations between two adjacent tetrahedral centres. Schematic drawings of (**c**) ZB and (**d**) WZ crystals with tetrahedral (*T*_*d*_) and hexagonal (*C_3v_*) symmetries, respectively, showing the spatial orientations of the analogous 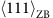 and 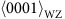 lattice directions. (**e**,**f**) DFT-simulated absorption spectra and imaginary part of dielectric function (Im(*ɛ*)) for bulk CdSe with (**e**) ZB and (**f**) WZ crystal phases. Im(*ɛ*) spectra are decomposed into two components indicated in **g** and **h**. The BZ is shown for (**g**) ZB and (**h**) WZ crystals. Yellow-coloured cylindrical regions in each BZ indicate points along four Λ (Γ to L) directions in ZB and points along Δ (Γ to A) direction in WZ crystals that are structural analogues between the two crystals. Yellow spectra in panels **e** and **f** derive from these regions, and blue spectra derive from all other points.

To understand the origin of the phase sensitivity of *E*_1_ transitions and phase insensitivity of *E*_0_ transitions, we first describe the two crystal structures and then evaluate the electronic wavefunctions, focusing on bulk crystals. As shown in Fig. 5a,b, ZB and WZ structures of CdSe have identical tetrahedral coordination between nearest neighbour atoms, with four Cd–Se bonds per atom. Between the two structures, there is only a small difference in bonding energy and bond length resulting from interactions with second nearest neighbours due to how bonds are arranged in either staggered or eclipsed geometries^31^. In ZB, all bonds are in the staggered conformation, resulting in a high degree of three-dimensional symmetry (*T*_*d*_ point group) with four equivalent lattice directions parallel to each bond direction, denoted [111]_ZB_, 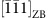, 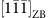 and 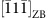 using cubic unit cell notation (see Fig. 5c). In WZ, one of the four bonds connected to each atom is in an eclipsed conformation and all such eclipsed bonds are parallel in the lattice. This single conformational difference gives the WZ structure a less symmetric lattice (*C_3v_* point group) in which the direction of eclipsed bonds constitutes a unique [0001]_WZ_ axis (the *c* axis) in a hexagonal unit cell (see Fig. 5d). The WZ structure thus lacks additional directions of bond axis symmetry, resulting in substantial differences in bonding electron energy levels compared to ZB. This leads to crystal-field splitting, for example, of the uppermost valence-band energy states. As we describe next, the magnitude in differences due to phase depends on the energy and momentum of the electron undergoing an optical transition in the crystal, and thus determines how *E*_0_ and *E*_1_ peaks depend on phase.

### Phase-dependent absorption spectra of bulk CdSe

For CdSe, subtle bonding differences between ZB and WZ result in only small differences in bandgap *E*_0_ transitions (*E*_0,WZ_=1.74 eV; *E*_0,ZB_=1.76 eV)^63^. As shown in Fig. 5e,f, DFT absorption spectra of bulk CdSe are similar for ZB and WZ at energies from *E*_0_ to ∼4 eV, consistent with experimental NC spectra and DFT simulations of NCs. Also consistent with NCs, bulk ZB and WZ absorption spectra differ substantially at higher energies (4–6 eV). These *E*_1_ critical points are well-known in experimental spectra of bulk CdSe and other direct bandgap semiconductors^47^,^64^, with slightly shifted energies compared with NCs, ∼4.5 eV for ZB and ∼5.1 eV for WZ. Spectra in Fig. 5 were calculated within the independent quasiparticle approximation (neglecting excitonic effects). We also calculated the spectra with excitonic effects and observed that spectral features were similar, although with slightly red-shifted energies (Supplementary Fig. 9), justifying our use of exciton-free simulations of NCs, for which including excitons in calculations is still intractable. It is also noteworthy that accurate quasiparticle energies can be computed using many-body perturbation theory^65^; however, due to large computational costs, we simulated those here using a scissor operator that corrects the fundamental bandgap to the experimental value.

To determine the *E*_1_ peak origin, we decomposed spectra based on reciprocal space contributions. The first Brillouin zone (BZ) is the primitive unit cell for the entire crystal in reciprocal space, shown for each polymorph in Fig. 5g,h. Yellow cylinders indicate directions of high crystalline symmetry that are structural analogues between ZB and WZ. For ZB the four 〈111〉_ZB_ directions correspond to the Λ_ZB_ direction (Γ to L) and for WZ the single 〈0001〉_WZ_ direction corresponds to the Δ_WZ_ direction (Γ to A). With this breakdown, peak origins are clear in decomposed spectra, shown in Fig. 5e,f. *E*_0_ transitions, and almost all spectral features at energies less than 4 eV for both structures, derive from analogous Λ_ZB_ and Δ_WZ_ directions. *E*_0_ transitions arise entirely from the BZ zone centre (Γ point) in direct bandgap semiconductors. The yellow region, corresponding to ∼28% of the ZB and WZ BZs, yields similar decomposed spectra for both phases at all energies, except in the *E*_1,ZB_ energy region. The *E*_1,ZB_ peak clearly arises from this shared symmetry and this peak is also present at the same energy in WZ, but it is substantially attenuated and contributes just a minor shoulder in the spectrum. On the other hand, the distinguishable *E*_1,WZ_ peak has a different origin deriving from the rest of the BZ, independent from Λ_ZB_ and Δ_WZ_ directions. ZB also has high energy peaks arising from these other directions of symmetry; however, they are at energies higher than what can be measured with ultraviolet–visible absorption spectroscopy due to overlap with solvent absorption. These findings are largely consistent with previous spectroscopic ellipsometry studies of bulk CdSe in ZB and WZ phases^47^, as well as representations of electronic band structures along high-symmetry directions and the corresponding joint density of states (Supplementary Fig. 10).

These origins are also helpful for explaining why *E*_1_ transitions exhibit less size dependence compared with *E*_0_ transitions. The *E*_0_ peak derives from the Γ point, which exhibits high curvature in the electronic band dispersion curve (Supplementary Fig. 10). High curvature causes the charge carriers involved in the transition to have small effective masses and therefore large sizes that are susceptible to energy shifts due to crystal size (quantum confinement). Both types of *E*_1_ transitions derive from higher energy dispersion bands, which are relatively flat, leading to smaller charge carriers and weaker quantum confinement^66^. However, the *E*_1,ZB_ peak derives from bands in Λ_ZB_ directions that are more curved than the higher energy bands contributing to the *E*_1,WZ_ peak, explaining why the *E*_1,ZB_ peak exhibits some size dependence. In addition, *E*_1,ZB_ peak energies in Fig. 1d arise from many more electronic transitions compared with band-edge transitions, so their values cannot be explained as a single transition. In particular, higher energy *E*_1,WZ_ peaks derive from bands over a broader range of energies; thus, quantum confinement effects that pare away contributing transitions may be less evident compared with those for ZB, for which contributing bands have closer energies. An additional contribution may come from mathematical extraction of the *E*_1_ transition values through second derivative spectra, which loses accuracy when evaluating highly convolved peaks^49^.

### Wavefunction analysis

**Figure 6:**
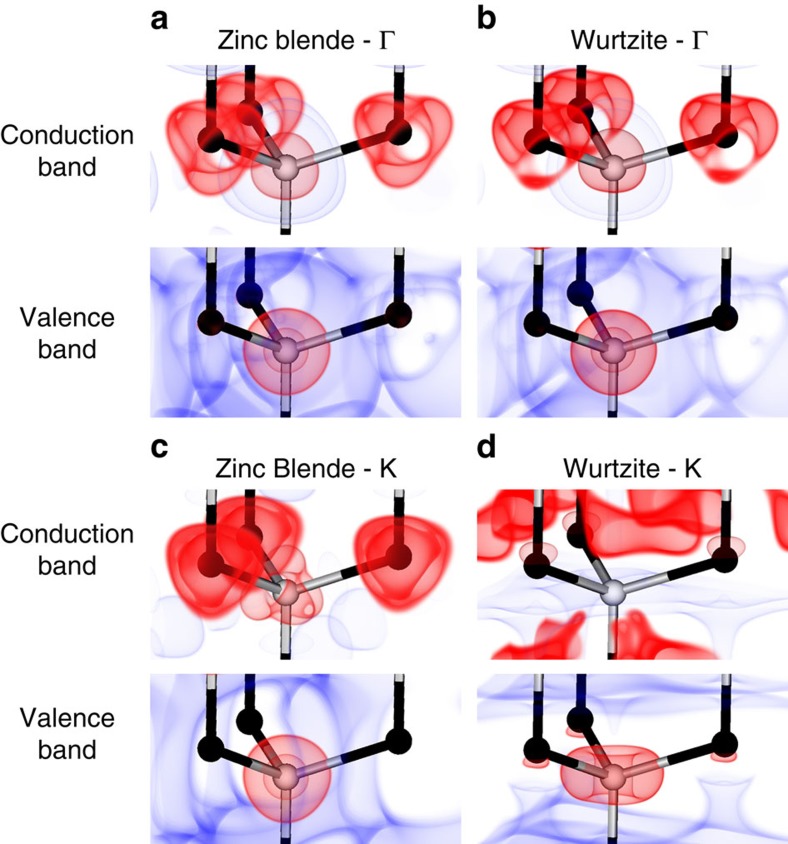
Wavefunctions of electronic energy states in bulk ZB and WZ CdSe. Atoms and bonds are shown in ball-and-stick format with Cd in black and Se in grey. Isosurfaces are shown for the top 95% of the squared wavefunction in red to depict high localization and the lowest 5% in blue to depict nodes. Images are shown for the *E*_0_ transition wavefunctions which are (**a**) ZB valence and conduction bands at the Γ point and (**b**) WZ valence and conduction bands at the Γ point, showing that the two are nearly identical. Here, the WZ valence band includes the split-off energy band to equalize the number of energy states to that of ZB, which intrinsically has higher degeneracy. Wavefunctions are also shown for higher energy analogous points in the BZ: (**c**) ZB and (**d**) WZ valence and conduction bands at K points. For these latter two examples, drastically different wavefunction localizations yield drastically different transition energies.

To depict the origins of these two transitions at the atomic scale, rather than crystalline scale, we used DFT to calculate Kohn–Sham wavefunctions of different electronic energy bands of ZB and WZ CdSe in bulk. All *E*_0_ and *E*_1_ peaks derive primarily from electronic transitions from valence bands composed of Se (4*p*) bonding orbitals to conduction bands derived from Cd (5*s*) anti-bonding orbitals. This means that valence band-edge states are primarily localized on selenium atoms and conduction band states are primarily localized on cadmium atoms. Figure 6a,b show wavefunctions of the band-edge (Γ point) valence and conduction band for ZB and WZ, respectively, with red colours indicating high wavefunction localization (95th percentile) and blue colours indicating near-zero wavefunction localization (5th percentile; nodes). It is clear that selenium atoms are the primary location for the valence band and cadmium atoms are the primary location for the conduction band states. Importantly, despite differences in bonding geometry, the wavefunctions for both phases appear nearly identical at band-edge Γ points, meaning that there is little interaction beyond the nearest neighbor atoms underlying these electronic energy levels, explaining why the *E*_0_ transitions are similar for WZ and ZB. However, at other analogous locations of the two BZs, the wavefunctions are highly dissimilar, as depicted in Fig. 6c,d for analogous K points. For WZ, these wavefunctions show strong polarization along the *c* axis that arises from more distant interactions that shift the transition energies and oscillator strengths.

### Origins of *E*_1_ depletion in polytypic NCs

The lattice periodicity difference may explain the observed trend of reduction in total *E*_1,WZ_ oscillator strength for polytypic NCs compared with pure-phase NCs. The *E*_0_ transitions and the region up to 4 eV were relatively unaffected by polytypism (Fig. 3b), which should be expected due to the nearly identical wavefunctions contributed by both ZB and WZ in the low energy electronic energy levels. Therefore, the *E*_1,ZB_ peak should likewise be unaffected, because it arises from similar transitions in both ZB and WZ from their analogous Λ_ZB_ and Δ_WZ_ directions (Fig. 5e,f). This is consistent with our experimental results, as we find that *E*_1,ZB_ was not depleted in polytypic NCs (Fig. 3f,g). Instead, the *E*_1,WZ_ peak was extensively depleted in both the experimental and theoretical spectra. This is logical because the *E*_1,WZ_ peak derives from non-analogous regions of the two phases, so a smaller number of atoms contribute to its total oscillator strength in a mixed phase NC. Therefore, the *E*_1,WZ_ peak effectively derives from a smaller NC compared with the *E*_1,ZB_. The result of this should be an attenuation of WZ-specific oscillator strength because the relative strength of the *E*_1_ peaks in the spectrum strongly depend on size (shown in Fig. 1c).

### Other materials

All results shown above are for quasi-spherical CdSe NCs. However, we also observed that phase-specific *E*_1_ energy trends hold for rod-like WZ NCs and flat nanoplatelets with WZ or ZB crystal structures (Supplementary Fig. 11). However, the absolute values of *E*_1_ energies can be shifted from those of spherical NCs. We are further investigating these shape-specific trends to determine the quantitative impact of aspect ratio and confinement dimensions. To determine if these findings can be similarly used for optical crystallography of other polymorphic materials, we synthesized additional NC compositions and performed DFT analysis on other materials. DFT spectra of bulk CdS and CdTe similarly show phase-distinguishing *E*_1_ peaks that arise from transitions analogous to those in CdSe, but with shifted energies depending on the chalcogen (Supplementary Fig. 12). We also verified experimentally that *E*_1_ transitions differ for *E*_0_-matched WZ and ZB CdS (Supplementary Fig. 13), but syntheses of phase-pure CdS are not as advanced as those for CdSe, yielding different degrees of inhomogeneous broadening. Notably, we were only able to synthesize CdTe in pure-ZB or polytypic phases using literature methods and could not obtain pure-WZ NCs. It is further likely that *E*_1_ transitions will not only allow solution-based phase distinction of CdE materials, but also ZnE and HgE materials, which show robust *E*_1_ critical point peaks in spectroscopic ellipsometry measurements of NCs on solid substrates^67^,^68^. It will also be interesting to evaluate higher energy transitions in I–III–VI_2_ materials such as CuInS_2_ and CuInSe_2_, which similarly exhibit polymorphism and polytypism between hexagonal and cubic phases^69^. However, in the cubic phase of these materials, Cu^+^ and In^+^ ions can either be ordered or disordered in the unit cell, yielding XRD patterns that are difficult to distinguish, such that complementary crystallographic methodologies may be of great value.

## Discussion

Through both theoretical and experimental evidence, we established that high-energy absorption features (*E*_1_ peaks) in II–VI NCs can unambiguously distinguish crystal phase. This confers the new capability to analyse and characterize the phase of very small NCs and polytypic NCs where powder XRD cannot provide clear crystal phase information. More importantly, this provides a new and powerful ability to continuously measure phase during synthesis or processing in solution by absorption spectroscopy, which can be more simple, rapid, high-throughput and potentially more accurate for structural characterization compared with solid-phase diffractometry. Moreover, optical absorption spectra of NCs are rich in information when samples are homogeneous, exhibiting discrete atom-like electronic transitions, which is the source of their moniker ‘artificial atoms.' These spectra may thus contain more information than size and phase, and may be used to further reconstruct NC shape, surface facets and defects.

## Methods

### NC synthesis

NCs were synthesized using literature methods with minor modifications. ZB CdSe NCs were synthesized by the method of Chen *et al*.^34^ by heating a solution of a cadmium alkylcarboxylate, selenium dioxide, 1,2-hexadecanediol and 1-octadecene to 220–230 °C. ZB CdS NCs were synthesized using the same method, except selenium dioxide was replaced with elemental sulfur. WZ CdSe NCs were prepared by the method of Carbone *et al*.^33^ by injecting a solution of selenium in trioctylphosphine and diphenylphosphine into a reaction mixture of cadmium octadecylphosphonate, TOPO and trioctylphosphine at 300–365 °C. WZ CdS NCs were synthesized using the same method, except the selenium solution was replaced with a solution of bis(trimethylsilyl)sulfide in TBP. Polytypic CdSe NCs were synthesized using the method of Qu *et al*.^61^ by injecting a solution of selenium in trioctylphosphine into a reaction mixture of cadmium oleate, TOPO and hexadecylamine at 320 °C. Reagents used in NC synthesis are described in Supplementary Note 1 and detailed synthetic conditions are provided in Supplementary Note 2 and Supplementary Tables 1 and 2.

### Photochemical etching

Photochemical etching of ZB CdSe NCs was performed using the methods of Lim *et al*.^62^ CdSe NCs were dispersed in chloroform (∼3 ml, absorbance ∼3 at the first exciton peak) and OLA (∼0.5 ml) in a 4 ml quartz cuvette with a small magnetic stir bar. Etching was initiated by 254 nm illumination with a 4W lamp (UVP UVGL-25 hand-held UV lamp) with rapid stirring. The reaction was monitored by measuring absorption spectra every 5–10 min to observe a continuous spectral blue-shift and a broadening of the band-edge absorption transitions, indicative of an increase in size distribution. When the absorption spectrum reached the desired wavelength, aliquots were removed and NCs were purified by precipitation with excess methanol (∼10 × excess of aliquot volume) with a few drops of OAc. Purified NCs were redispersed in hexane for analysis.

### Absorption spectroscopy

Samples were prepared by dispersing pure NCs in hexane or water in a quartz cuvette. Absorption spectra were acquired between 200–800 nm in 1 nm steps using an Agilent Cary 5000 ultraviolet–visible–near-infared spectrophotometer. Samples were diluted so that there was no absorption signal saturation over the full spectral range. Absorption values in wavelength scale, *A*(*λ*), were converted to absorption values in energy scale, *A*(*E*), by^48^:


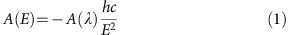


where *h* is Planck's constant and *c* is the speed of light and the negative sign indicates the increase in wavelength corresponding to a decrease in energy. *E*_0_ and *E*_1_ energies were measured as the local minima in the second derivative of these energy-scale spectra.

### Powder XRD

Samples for XRD were prepared by dropping and drying highly concentrated hexane dispersions of pure NCs on glass slides. XRD patterns were acquired using a Bruker/Siemens D-5000 X-ray diffractometer in the Frederick Seitz Materials Research Laboratory Central Research Facilities at the University of Illinois. The instrument was equipped with a Cu K-*α* line (*λ*=1.5418 Å) X-ray source operating at 40 kV, 30 mA and scattering signals were collected in *θ*–2*θ* scan mode in 15°–75° 2*θ* range with 2° min^−1^ scanning speed.

### XRD simulations

To fit the empirical XRD pattern of polytypic CdSe NCs in Fig. 3a, simulated XRD patterns were generated by using previously published methods^28^. CdSe NCs were simulated as quasi-spherical shapes (*d*=3.6 nm) with bulk lattice constants containing 11 CdSe layers along the [0001]_WZ_=[111]_ZB_ direction. Polytypic structures were simulated by inserting different numbers of stacking faults in these layers, giving 2^10^=1,024 possible structures. XRD patterns of each of these structures were simulated in the 15–75° 2*θ* range with 0.1° steps. Then, using these simulated patterns as a basis set, the empirical XRD pattern was fit using a least-square fitting algorithm. In a preliminary fitting using all 1,024 simulated patterns as basis, 53 patterns were found to have >0.2% contribution to simulate the pattern closest to the empirical one. The final fitting was performed with these 53 simulated patterns.

### Phase-dependent trends in *E*_1_ spectra

The phase-dependent optical trend for polytypic CdSe NCs was determined using the ratio of absorption values at *E*_1,ZB_ and *E*_1,WZ_, *A*(*E*_1,WZ_)/*A*(*E*_1,ZB_), measured from experimental spectra for *E*_0_-matched NCs (three spectra in Fig. 3b), as well as theoretical spectra (five spectra in Fig. 3d). For experimental spectra, the energy values of *E*_1,ZB_ and *E*_1,WZ_ were measured from the peaks derived from phase-pure ZB or WZ NC spectra, respectively, and then the absorption ratio was linearly normalized so that the value for pure WZ (100% WZ) was 0 and the value for pure ZB (0% WZ) was 1. Then, using the same method, energy values and normalization parameters, the absorption peak ratio for the polytypic NC was calculated and normalized, and its percentage WZ was defined by XRD simulations. The same procedure was applied to the theoretical spectra, except the energy values of *E*_1,ZB_ and *E*_1,WZ_ were measured from the theoretical phase-pure ZB or WZ spectra, rather than the experimental spectra.

### *Ab initio* calculations

We used DFT^50^,^70^ to compute Kohn–Sham states and eigenvalues as starting electronic structures for the solution of the Bethe–Salpeter equation. Exchange and correlation were described using the generalized-gradient approximation by Perdew, Burke and Ernzerhof^51^, and the electron–ion interaction was described by the projector-augmented wave method^71^. Wave functions were expanded into a plane-wave basis up to a cutoff energy of 520 eV (400 eV for NCs). For bulk WZ and ZB CdSe, we used the atomic geometries provided by the Materials Project^72^ and all atomic geometries of CdSe NCs were relaxed until the Hellman–Feynman forces were below 10 meV Å^−1^. To correct the DFT band-gap error due to the neglect of quasiparticle effects, we used a scissors operator that rigidly shifts the conduction bands to open up the bandgap to 1.76 eV (WZ) and 1.74 eV (ZB), respectively^63^. We verified that this approach accurately described higher energy bands as well as their dispersion by comparing to data computed using the HSE06 hybrid exchange-correlation functional^73^ (see Supplementary Fig. 3). To achieve an accurate description of optical properties (including excitonic and local-field effects) from first principles, we solved the Bethe–Salpeter equation for the optical polarization function^65^. After constructing the excitonic Hamiltonian that includes statically screened Coulomb attraction and unscreened exchange terms, the dielectric function was computed using a time-propagation technique^74^. To converge these optical spectra with respect to BZ sampling, we used 8 × 8 × 6:7 × 7 × 5:19.4 × 19.4 × 15.6 (bulk WZ) and 10:5:30 (bulk ZB) hybrid *k*-point meshes^75^ and applied a small random shift to lift degeneracies. For NCs we used 2 × 2 × 2 Gamma-centred Monkhorst–Pack *k*-point grids^76^ and did not take excitonic effects into account due to the large computational cost. All calculations were carried out within the Vienna *Ab-initio* Simulation Package^53^,^54^ and the Bethe–Salpeter equation implementation discussed in previous reports^75^,^77^.

### Data availability

Data supporting the experimental results and conclusions of this article are available from A.M.S. upon request. Data from theoretical simulations are available from A.S. at schleife@illinois.edu.

## Additional information

**How to cite this article:** Lim, S. J. *et al*. Optical determination of crystal phase in semiconductor nanocrystals. *Nat. Commun.* **8,** 14849 doi: 10.1038/ncomms14849 (2017).

**Publisher's note**: Springer Nature remains neutral with regard to jurisdictional claims in published maps and institutional affiliations.

## Supplementary Material

Supplementary Information Supplementary Tables, Supplementary Figures, Supplementary Notes and Supplementary References
