# Supplementary material for: Optical determination of crystal phase in semiconductor nanocrystals
Source: Nat Commun. 2017 May 17;8:14849. doi: 10.1038/ncomms14849 (PMC5442309; doi:10.1038/ncomms14849)
Supplement: Supplementary Information — Supplementary Tables, Supplementary Figures, Supplementary Notes and Supplementary References [file ncomms14849-s1.pdf]

Supplementary Table 1. Synthetic conditions for zinc blende CdSe nanocrystals

E_0 (eV)	Precursors	Growth temp (°C)	Growth time (min)	2nd Growth
2.70	$Cd(BAc)_2$, SeO_2 (Cd:Se = 2:1)	220	1	No
2.30	$Cd(BAc)_2$, SeO_2 (Cd:Se = 1:1)	230	15	No
2.16	$Cd(BAc)_2$, SeO_2 (Cd:Se = 2:1)	230	15	Add TOPSe[a]
2.09	$Cd(MAc)_2$, SeO_2 (Cd:Se = 1:1)	230	15	No
2.04	$Cd(SAc)_2$, SeO_2 (Cd:Se = 2:1)	230	15	Add TBPSe[b]

[a] Selenium in trioctylphosphine, 0.1M
[b] Selenium in tributylphosphine, 0.5M

Supplementary Table 2. Synthetic conditions for wurtzite CdSe nanocrystals

E_0 (eV)	Injection temp (°C)[a]	DPP stock (μL)[b]	Growth time (s)[a]
2.70	300	100	0
2.30	370	20	15
2.16	360	10	60
2.09	365	10	40
2.04	365	10	50

[a] Se solution was injected at the injection temperature. After the growth time, the heating mantle was removed and the solution was rapidly cooled with an air stream to quench nanocrystal growth.
[b] Diphenylphosphine in trioctylphosphine, 0.8M

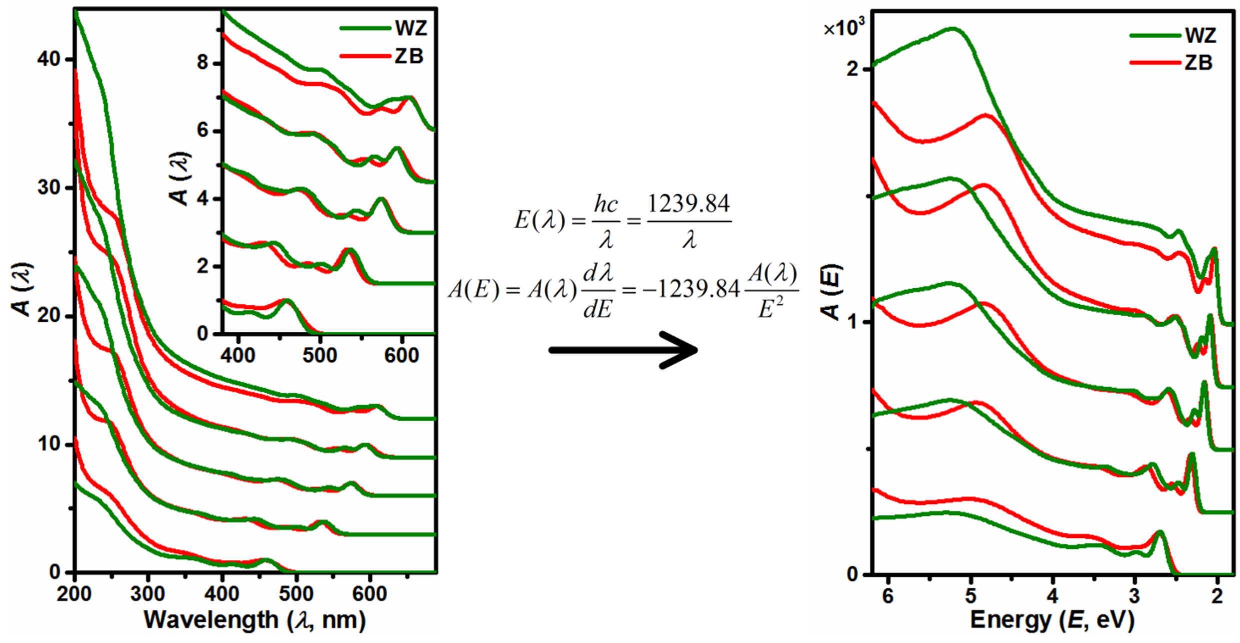

Supplementary Figure 1. Jacobian transformation of absorption spectra. Each CdSe nanocrystal absorption spectrum was transformed from a function of wavelength ($A(\lambda)$, left) to a function of energy ($A(E)$, right). WZ = wurtzite; ZB = zinc blende.

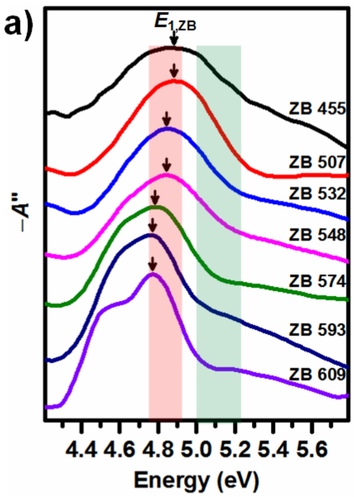

Supplementary Figure 2. Size-dependent E_1 energies of CdSe nanocrystals. Second derivative energy-scale absorption spectra are shown for (a) ZB and (b) WZ nanocrystals with different sizes in the region of E_1 peaks. Zero-derivative spectra are shown in Figure 1c in the main text. Arrows point the E_1 peak energies determined from the maxima. Red and green shades indicate the energy ranges of $E_{1,ZB}$ and $E_{1,WZ}$ peaks, respectively, which are clearly separated. WZ = wurtzite; ZB = zinc blende.

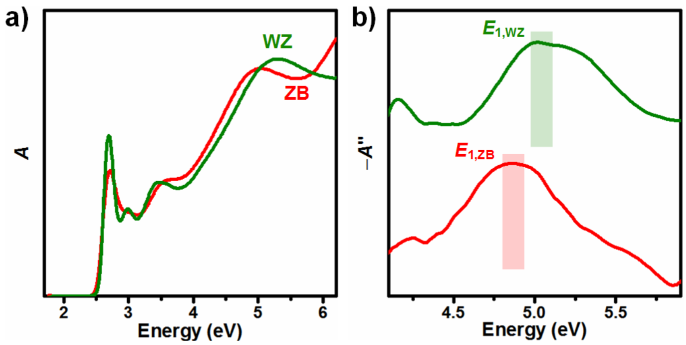

Supplementary Figure 3. Phase-dependent E_1 energies of 2-nm CdSe nanocrystals. (a) Energy-scale absorption spectra and (b) their second derivatives for 2-nm CdSe nanocrystals with ZB (red) and WZ (green) phases. Red and green shades in (b) indicate the energy ranges of $E_{1,ZB}$ and $E_{1,WZ}$ peaks, respectively, which are clearly separated. WZ = wurtzite; ZB = zinc blende.

.

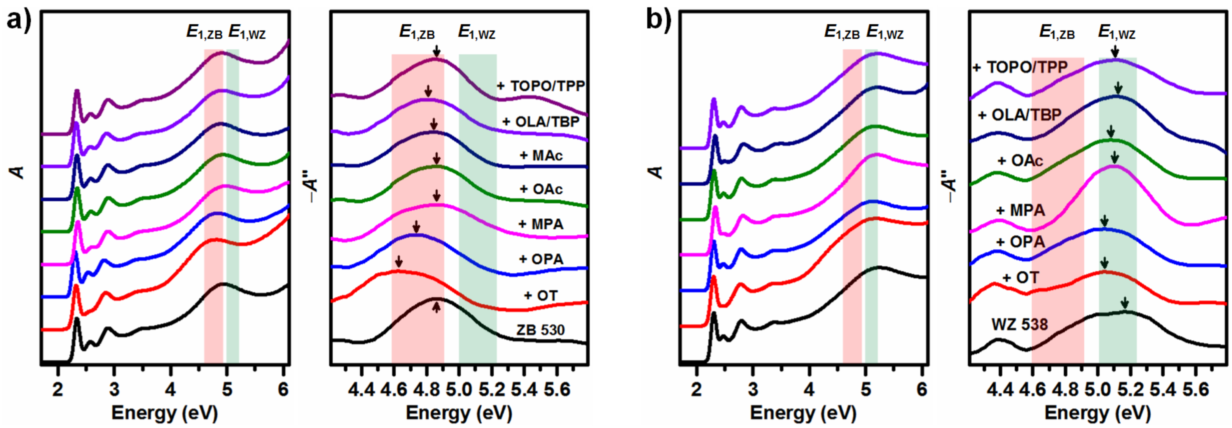

Supplementary Figure 4. Ligand-dependent E_1 energies of CdSe nanocrystals. Absorption spectra (left) and their second derivatives (right) of CdSe nanocrystals with (a) ZB and (b) WZ phases. Nanocrystals were surface modified with a variety of ligands. Red and green shades respectively indicate the energy ranges of $E_{1,ZB}$ and $E_{1,WZ}$ peaks, showing that the two region do not overlap even with some ligand-dependent band broadening. All absorption spectra were measured in hexane, except those coated with mercaptopropionic acid ligands, which were measured in aqueous 2mM NaOH. OT = octanethiol; OPA = octylphosphonic acid; MPA = mercaptopropionic acid; OAc = oleic acid; MAc = myristic acid; OLA = oleylamine; TBP = tributylphosphine; TOPO = trioctylphosphine oxide; TPP = triphenylphosphine; WZ = wurtzite; ZB = zinc blende.

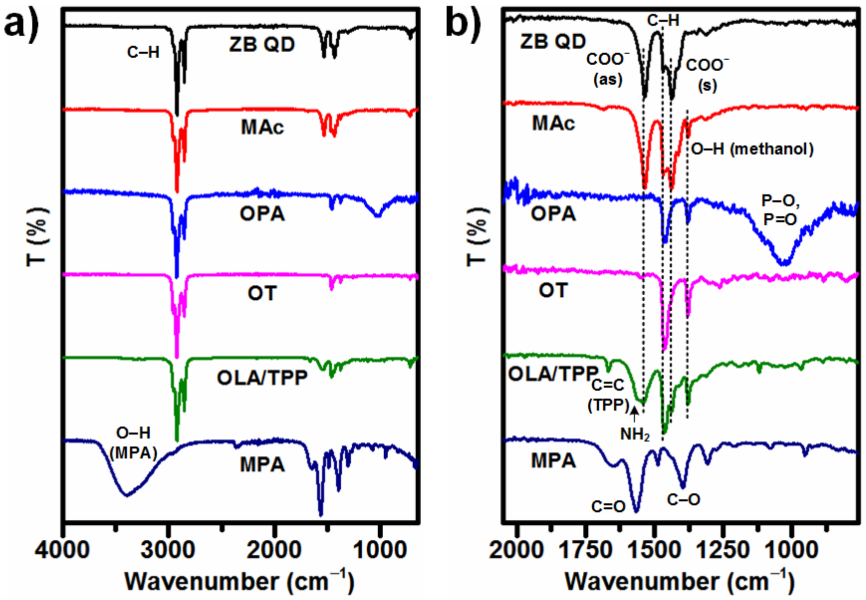

Supplementary Figure 5. FT-IR spectra of surface-modified ZB CdSe nanocrystals. Spectra are shown as (a) full range (650–4,000 cm^{-1}) and (b) zoom-in (650–2,000 cm^{-1})) of ZB CdSe nanocrystals before (black) and after (coloured) surface modification with various ligands. Spectra confirm the exchange of ligands by the characteristic vibrational peaks of functional groups present on the different ligands. Spectra show that as-synthesized nanocrystals (ZB QD) have carboxylates, nanocrystals modified with myristic acid (MAc) have carboxylates, nanocrystals modified with octylphosphonic acid (OPA) have phosphonates but no carboxylates, nanocrystals modified with octanethiol (OT) have alkanes but no carboxylates, and nanocrystals modified with oleylamine/triphenylphosphine (OLA/TPP) have carboxylates, alkenes (phenyl from TPP), and amines. The nanocrystals modified with mercaptopropionic acid (MPA) have carboxylic acids present, but none of the carboxylates peaks from the original ligands remain. No characteristic peak of thiol or thiolates is detected in the OT- and MPA-modified nanocrystal spectra due to the limited spectral range (C–S vibration: ~600 cm^{-1}). OLA/TPP-modified nanocrystal spectra show that these L-type ligands cannot fully displace the original X-type carboxylates. Methanol was used for purification, and its characteristic peak is present in some samples. WZ = wurtzite; ZB = zinc blende.

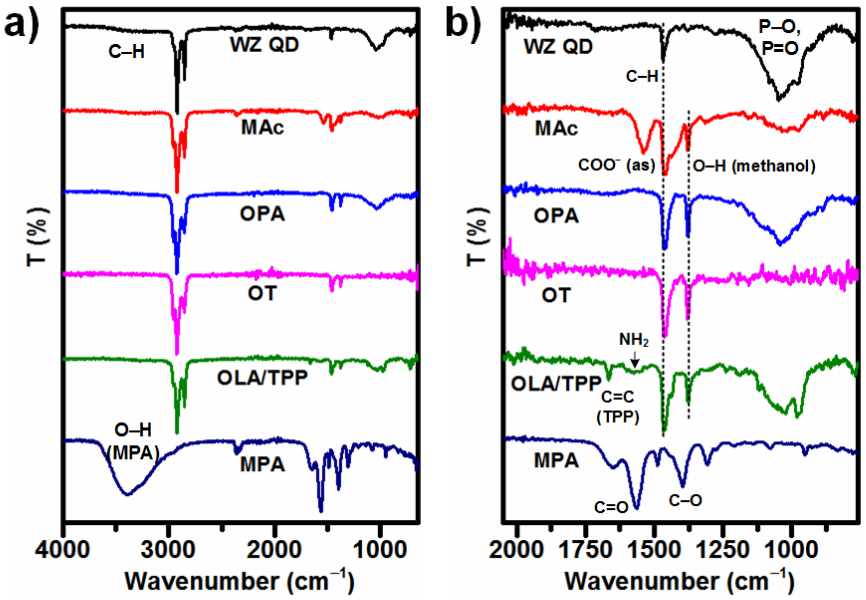

Supplementary Figure 6. FT-IR spectra of surface-modified WZ CdSe nanocrystals. Spectra are shown as (a) full range (650–4,000 cm^{-1}) and (b) zoom-in (650–2,000 cm^{-1})) of WZ CdSe nanocrystals before (black) and after (coloured) surface modification with various ligands. Spectra show that as-synthesized nanocrystals (WZ QD) have phosphonates, nanocrystals modified with myristic acid (MAc) have carboxylates with some residual phosphonates, nanocrystals modified with octylphosphonic acid (OPA) have phosphonates, nanocrystals modified with octanethiol (OT) have alkanes but no phosphonates, and nanocrystals modified with oleylamine/triphenyl-phosphine (OLA/TPP) have phosphonates, alkenes (phenyl from TPP), and amines. The nanocrystals modified with mercaptopropionic acid (MPA) have carboxylic acids present, but none of the phosphonate ligands remain. Methanol was used for purification, and its characteristic peak is present in some samples. WZ = wurtzite; ZB = zinc blende.

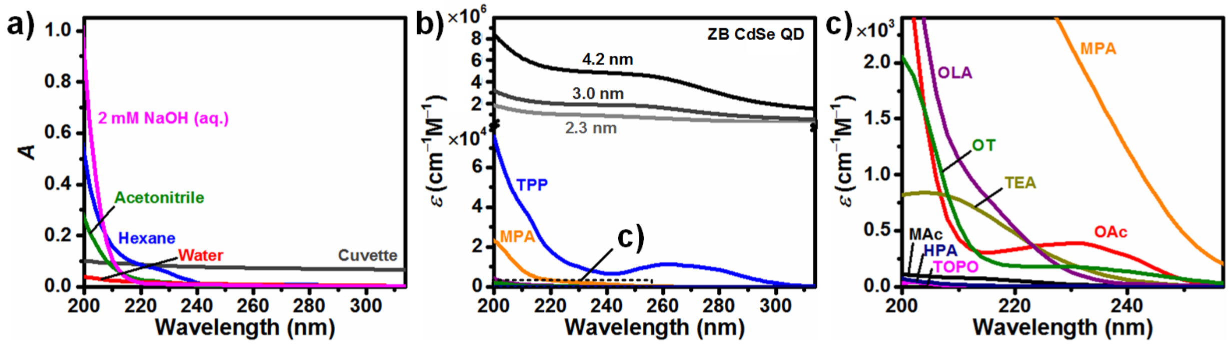

Supplementary Figure 7. Absorption spectra and extinction coefficients of solvents and ligands. (a) Absorption spectra of quartz cuvette (path length 5 mm; background: air) and various solvents (background: air + cuvette) suitable for measurements in the E_1 spectral region (attenuation coefficient < 2 cm^{-1} between 200–300 nm). (b) Comparison of molar extinction coefficients (ε) of CdSe nanocrystals (d = 2.3–4.2 nm, top) and organic ligands (bottom) in the ultraviolet spectrum. Absorption spectra of all nanocrystals and ligands were measured in hexane except for mercaptopropionic acid (MPA, in water) and hexylphosphonic acid (HPA, in acetonitrile). (c) Expanded view of the ε spectra of low-absorbing ligands with $\varepsilon < 2 \times 10^3$ $cm^{-1}M^{-1}$, which is 10^3–10^4 fold smaller than those of CdSe nanocrystals. Because the number of nanocrystal surface atoms is in the range of 90 (2.3 nm) to 330 (4.3 nm), and assuming that all surface atoms are passivated by ligands, the contribution of the organic ligands in the ultraviolet absorption of nanocrystal samples can be expected to be < 3% for the smallest (2.3 nm) nanocrystals and < 2% for the largest (4.2 nm) nanocrystals. WZ = wurtzite; ZB = zinc blende.

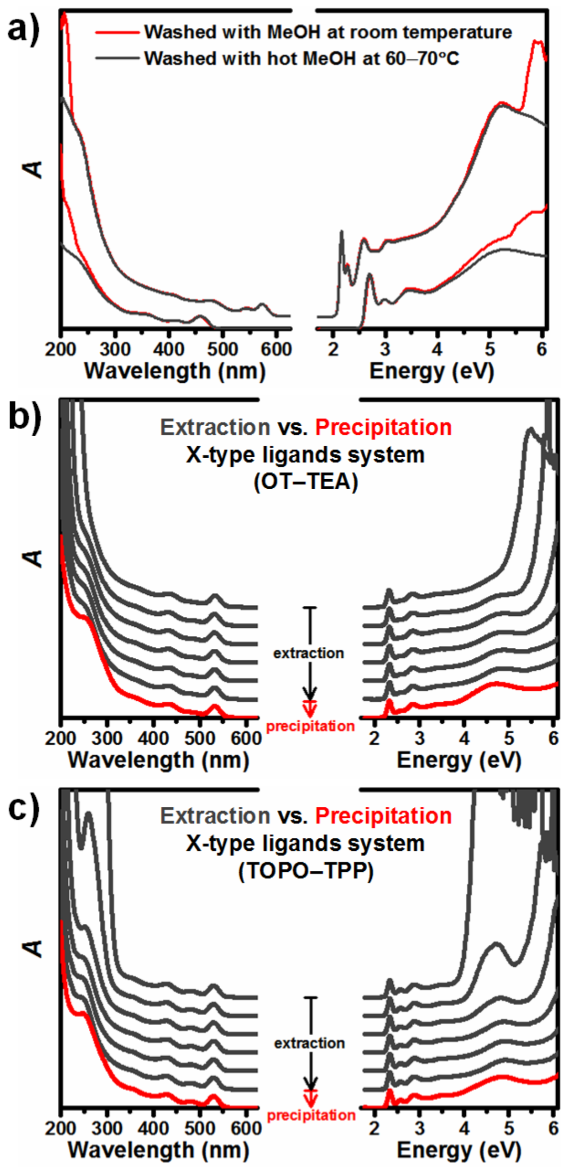

Supplementary Figure 8. Impact of the purification process on the high energy (E_1) absorption spectra of CdSe nanocrystals. (a) Absorption spectra (left: wavelength scale; right: energy scale) of two sizes of WZ CdSe nanocrystals in hexane after purification by washing with methanol at room temperature (red) and by washing with hot methanol, showing that hot methanol washing is more effective in removing residual byproducts (mostly cadmium phosphonate complexes) which absorb in the <250 nm spectral region. While this is critical for accurate measurement of E_1 peak energy, even partially impure WZ CdSe nanocrystals were accurately distinguishable from ZB nanocrystals in the E_1 spectrum. (b, c) Changes in absorption spectra of impure CdSe nanocrystals through purification via liquid–liquid extractions (grey) or nonsolvent precipitations (red). Examples are shown for nanocrystals with ~3,000-fold molar excess of (b) octanethiol (OT) or (c) trioctylphosphine oxide (TOPO)/triphenylphosphine (TPP) ligands in hexane during 5 successive extractions with acetonitrile (grey) followed by a precipitation with acetonitrile/acetone mixture and redispersion in hexane (red). Nearly identical spectra are obtained (compare red spectra) after purification, despite differing optical properties of the different ligands. WZ = wurtzite; ZB = zinc blende.

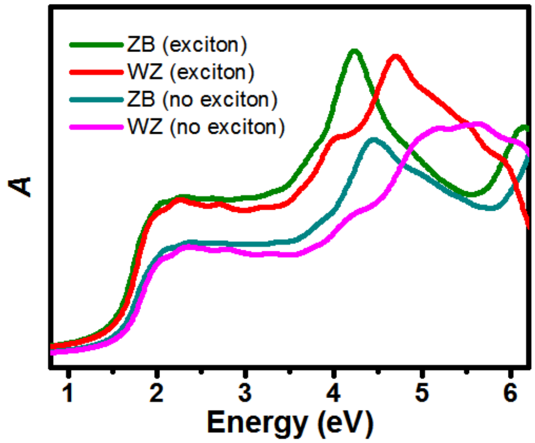

Supplementary Figure 9. Simulated absorption spectra of bulk CdSe. Optical absorption spectra for bulk WZ and ZB CdSe were computed using the independent quasiparticle approximation (no exciton) and the Bethe-Salpeter equation (BSE) approach that takes excitonic effects into account (exciton). The influence of excitonic effects can be seen as a redistribution of spectral weight. However, this effect is smaller than observed for many other semiconductors due to the large electronic screening in CdSe. This illustrates that the independent quasiparticle approximation can be used to describe the optical spectra of nanocrystals, for which the BSE approach becomes computationally unaffordable. WZ = wurtzite, ZB = zinc blende.

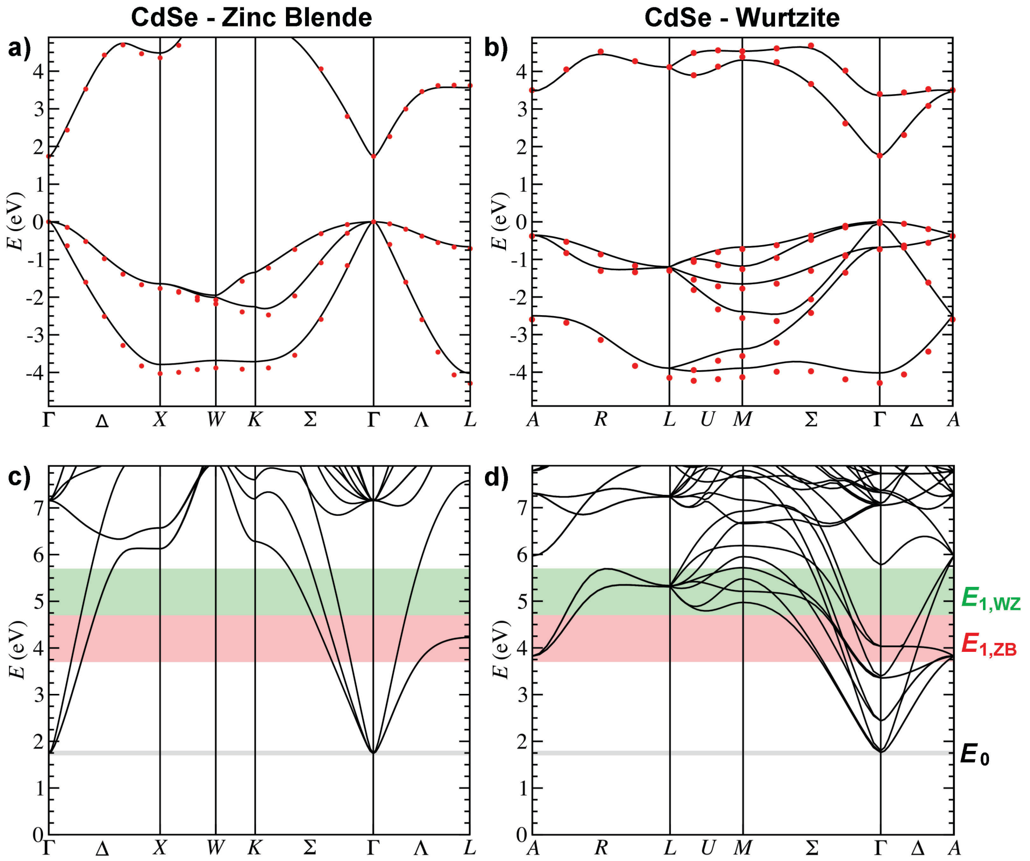

Supplementary Figure 10. Electronic energy levels in bulk CdSe. Electronic energy levels in bulk CdSe, for both zinc blende and wurtzite crystal structures, calculated using density functional theory (DFT). (a, b) Data are shown for zinc blende (a) and wurtzite (b), with solid lines showing DFT results and red dots indicating values computed using the HSE06 hybrid exchange-correlation functional (the gaps are shifted to experimental values in both cases). Both data sets agree well, justifying the use of the DFT+scissor approach to compute the electronic-structure for optical properties. (c, d) Joint band structure for each corresponding *k*-point for zinc blende (c) and wurtzite (d) are shown with indicated energy regions that contribute to the E_0, $E_{1,ZB}$, and $E_{1,WZ}$ transitions by colour codes. WZ = wurtzite; ZB = zinc blende.

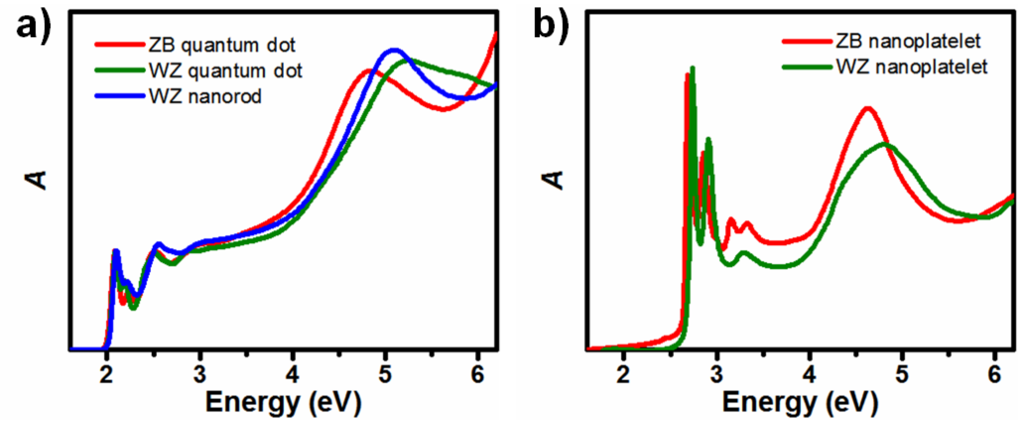

Supplementary Figure 11. Shape-dependent E_1 spectra of CdSe nanocrystals. (a) Absorption spectra of WZ CdSe nanorods (aspect ratio ~2) compare to those of spherical ZB and WZ CdSe nanocrystals with similar bandgap energies (~595 nm). (b) Absorption spectra of ZB and WZ CdSe nanoplatelets with similar bandgap energies (ZB 463 nm; WZ 450 nm). WZ = wurtzite; ZB = zinc blende.

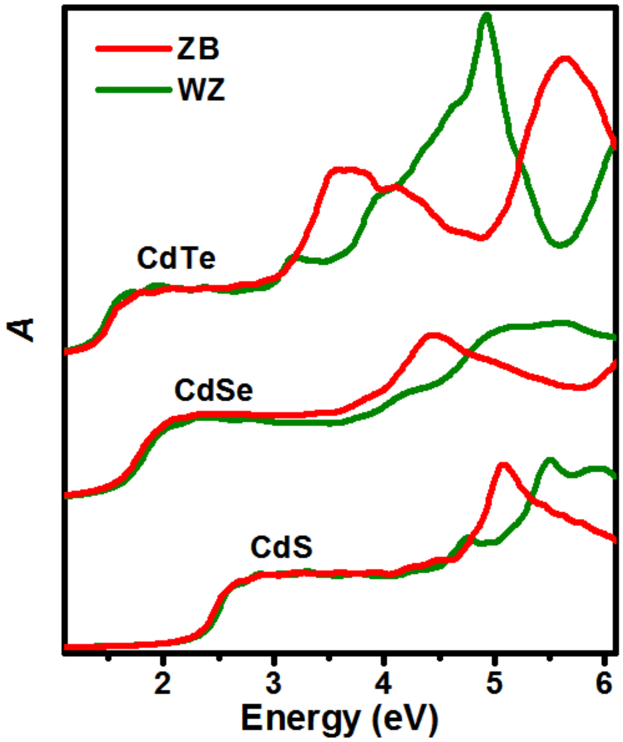

Supplementary Figure 12. Simulated absorption spectra of bulk cadmium chalcogenides. DFT-simulated absorption spectra are shown for bulk CdS, CdSe, and CdTe in zinc blende (ZB, red) and wurtzite (WZ, green) crystal phases.

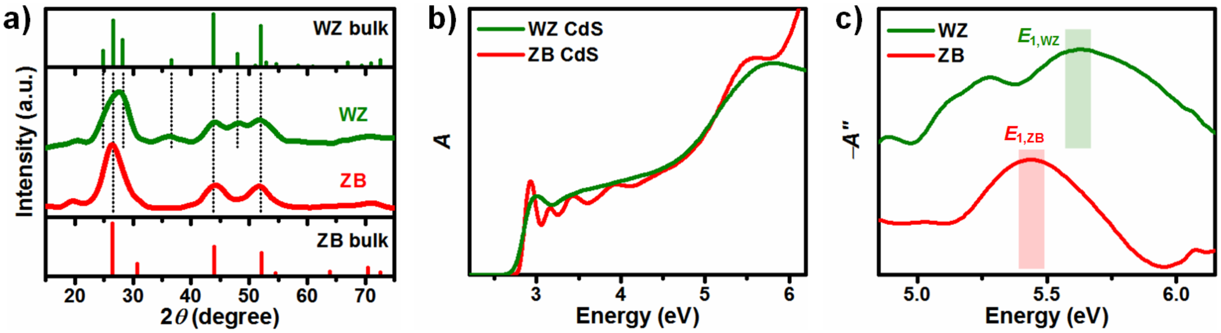

Supplementary Figure 13. Crystal phase-dependent XRD patterns and absorption spectra of CdS nanocrystals. (a) Powder XRD of zinc blende (ZB) and wurtzite (WZ) CdS nanocrystals. Coloured vertical lines represent the diffraction patterns for bulk ZB and WZ CdS. Dashed lines are guides for clarity. (b) Absorption spectra of ZB and WZ CdS nanocrystals in energy scale. (c) Second derivative spectra of ZB and WZ CdS nanocrystals in the E_1 energy region, showing distinct positions of E_1 energies of ZB ($E_{1,ZB}$ 5.44 eV, red shade) and WZ ($E_{1,WZ}$ 5.64 eV, green shade) CdS NCs.

Supplementary Notes

Supplementary Note 1: Chemicals

Cadmium oxide (CdO, 99.99+%), cadmium acetate hydrate ($Cd(Ac)_2 \cdot H_2O$, 99.99+%), selenium dioxide (SeO_2, ≥99.9%), selenium powder (Se, ~100 mesh, 99.99%), bis(trimethylsilyl)sulfide ($(TMS)_2S$, synthesis grade), stearic acid (SAc, 95%), tributylphosphine (TBP, 97%), triphenylphosphine (TPP, 99%), diphenylphosphine (DPP, 98%), hexadecylamine (HDA, 90%), 1-octanethiol (OT, ≥98.5%), 3-mercaptopropionic acid (MPA, ≥99%), 1,2-hexadecanediol (HDD, 97%), hexylphosphonic acid (HPA, 95%), trimethylamine (TEA, ≥99%), tetramethylammonium hydroxide (TMAOH, 25 wt. % in methanol) and sodium hydroxide (NaOH) were purchased from Sigma-Aldrich. Cadmium chloride anhydrous ($CdCl_2$, 99.99%) and octylphosphonic acid (OPA, 99%) was obtained from Alfa Aesar. 1-octadecene (ODE, 90% tech.), oleylamine (OLA, 80–90% C18-content), oleic acid (OAc, 90% tech.), and myristic acid (MAc, 99%) were purchased from Acros Organics. Behenic acid (BAc, 99%) was obtained from MP Biomedicals and octadecylphosphonic acid (ODPA, >99%) was purchased from PCI Synthesis. Trioctylphosphine oxide (TOPO, 99%) and trioctylphosphine (TOP, 97%) were acquired from Strem Chemicals. Solvents including chloroform, hexane, toluene, methanol, acetone, ethyl acetate, acetonitrile were purchased from various sources including Acros Organics, Fisher Scientific, Macron Fine Chemicals. All chemicals were used as purchased. Cd behenate ($Cd(BA)_2$), Cd stearate ($Cd(SAc)_2$), and Cd myristate ($Cd(MA)_2$) were synthesized and purified by following literature methods.[1] Sodium oleate (NaOAc) was prepared by titrating a OAc solution in methanol (0.1 M, 50 mL) with NaOH (0.1 M in methanol) to pH 8 at room temperature, then removing the solvent in a rotary evaporator. The product was rinsed with ice-cooled methanol then dried under vacuum.

Supplementary Note 2: Nanocrystal Synthesis

Zinc-blende (ZB) CdSe. ZB CdSe nanocrystals were prepared by the method of Chen *et al.*[1] In a typical synthesis, Cd carboxylate ($Cd(BAc)_2$, $Cd(SAc)_2$, or $Cd(MAc)_2$; 0.2–0.4 mmol), SeO_2 (0.2 mmol), HDD (0.2–0.4 mmol), and ODE (4–8 mL) were mixed in a 50-mL r.b.f. and dried under vacuum at ~100°C for 2 h. Then the temperature was raised to 220–230°C (220°C for d~2 nm, 230°C for larger nanocrystals) at a rate of ~20°C/min under nitrogen. The solution color changed from colorless to pale yellow at ~190°C, indicating CdSe nucleation. After reaching the growth temperature, the temperature was maintained for 15 min. The final NC size was larger when Cd carboxylates with shorter alkyl chain were used. If a 2nd growth was required, the selenium precursor solution (TOPSe 0.1M or TBPSe 0.5M) was added dropwise into the reaction mixture until the band-edge absorption reached the desired value. Detailed synthetic conditions for nanocrystals with different sizes are summarized in Supplementary Table 1. Nanocrystal growth was quenched by removing the heating mantle. When cooled to ~110°C, the reaction solution was mixed with chloroform (10 mL) containing OAc (1 mL) and OLA (0.6 mL). Purification was performed by precipitating the NCs with the addition of a mixture of methanol (15 mL) and acetone (15 mL). NC pellets were redispersed in hexane (~20 mL) and extracted twice with methanol (5–10 mL per cycle) followed by precipitating with excess methanol. Finally, NCs were washed with a few milliliters of acetone remove methanol, and dispersed in hexane as a stock solution.

For the synthesis of ZB CdSe nanocrystals with wurtzite-like band edge absorption features (ZB* nanocrystal in Figure 2), $Cd(BAc)_2$ (0.2 mmol), SeO_2 (0.2 mmol), HDD (0.2 mmol), ODE (4 mL), and NaOAc (0.2 mmol) were mixed in a 50-mL r.b.f., and dried under vacuum at ~100°C for 2 hours. Then the temperature was raised to 230°C at a rate of ~20°C min^{-1} under nitrogen. After reaching 230°C, the nanocrystals were grown for 15 min and the reaction was quenched by reducing the temperature.

Zinc-blende (ZB) CdS. ZB CdS nanocrystals were synthesized using a similar method to that of ZB CdSe. In a typical synthesis, $Cd(MAc)_2$ (0.2 mmol), S (0.2 mmol), HDD (0.2 mmol), and ODE (4 mL) were mixed in a 50-mL r.b.f., and dried under vacuum at ~100°C for 2 hours. Then the temperature was raised to 230°C at a rate of ~20°C/min under nitrogen. After reaching 230°C, the nanocrystals were grown for 15 min and the reaction was quenched by reducing the temperature. As-synthesized nanocrystals were purified by the same method used in ZB CdSe nanocrystals.

Wurtzite (WZ) CdSe. WZ CdSe nanocrystals were prepared by the method of Carbone *et al.*[2] In a typical synthesis, CdO (60 mg), ODPA (280 mg), and TOPO (3 g) were added to a 50-mL round bottom flask (r.b.f.) and dried under vacuum at ~100°C for 1 h. Then the mixture was heated to ~320°C under nitrogen until it became a clear colourless solution with the formation of a Cd-phosphonate complex. TOP (1 mL) was added and the temperature was stabilized at a specific temperature (300–365°C). A Se solution was prepared by dissolving Se powder (60 mg) in TOP (0.5 mL) with sonication then adding a specific amount of DPP (0.8 M stock in TOP, 10–100 µL). Nanocrystal nucleation was initiated by swiftly injecting the Se solution into the Cd solution, and the final size was tuned by controlling the injection temperature, amount of DPP, and the growth time. Detailed conditions are provided in Supplementary Table 2. As-synthesized nanocrystals were purified by diluting the reaction mixture with toluene (3 mL) while the mixture was still hot and not solidified (~100 °C) followed by precipitating the nanocrystals with excess methanol (~40 mL). After two more cycles of toluene–methanol washing, purified NCs were dissolved in hexane and stored as a stock solution. If absorption spectra show the presence of residual byproducts, NCs can be further purified by the addition of hot hexane–methanol (hexane:methanol 1:1 v/v) washing until the byproducts are completely removed (see Supplementary Figure 6a).

Wurtzite (WZ) CdS. WZ CdS nanocrystals were prepared by the method of Carbone *et al.*[2] In a typical synthesis, CdO (100 mg), ODPA (600 mg), and TOPO (3.3 g) were mixed in a 50-mL r.b.f., and dried under vacuum at ~100°C for 1 h. Then the mixture was heated to ~320°C under nitrogen until it became a clear colourless solution. A S precursor, prepared by dissolving $(TMS)_2S$ (200 µL) in TBP (3.7 mL), was rapidly injected into the Cd solution to initiate the CdS nucleation. The temperature was reduced to ~250°C for NC growth, and after 2min 30s the solution was rapidly cooled to ~100°C using a cool air stream. The as-synthesized nanocrystals were purified through the same protocol used for WZ CdSe NCs.

Polytypic CdSe. Polytypic CdSe nanocrystals were synthesized by the method of Qu *et al.*[3] with small modifications. A selenium precursor solution was prepared by dissolving Se powder in TOP (Se/TOP 0.4 M). A cadmium precursor solution was prepared by dissolving CdO (25.8 mg), OAc (810 µL), and ODE (8.7 mL) at ~200°C, followed by adding TOPO (1.8 g) and HDA (1.8 g). NCs were synthesized by injecting the selenium precursor into the cadmium precursor solution at 315°C. The solution was rapidly cooled ~45 s after injection to quench NC growth. The NCs were purified by precipitation with excess methanol (~40 mL) and two additional methanol washes. The purified NCs were dispersed in hexane for use.

Anisotropic CdSe. Synthetic protocols for nanorods and nanoplatelets were adopted from Lim *et al.*: reference 4 for WZ nanorods and reference 5 for ZB and WZ nanoplatelets.

Supplementary Note 3: Nanocrystal Ligand Exchange

Hydrophobic ligands. Purified ZB and WZ CdSe NCs (~1 nmol) in hexane (~2 mL) were mixed with a ~3,000-fold molar excess of various types of ligands, including OAc, MAc, OPA, OT, TOPO, TPP, OLA, and TBP at room temperature. A small amount of chloroform (0.5–1 mL) was added to make sure all ligands were well dissolved and the solution became homogeneous. When mixing with X-type (anionic) ligands such as carboxylic acid (OAc, MAc), phosphonic acid (OPA),

and thiol (OT), an excess of organic base (TEA) was added to deprotonate the ligands. The mixture was mixed at room temperature for 10–15 min. Then the NCs were precipitated by adding a mixture of polar solvents (acetonitrile/acetone/methanol), and the NC pellets were dried with acetone. The volume of methanol was minimized because due to its ability to excessively deplete bound ligands and thus aggregate NCs. Finally, purified NCs were dispersed in hexane for measurement of absorption spectra.

Hydrophilic ligands. ZB and WZ CdSe NCs pellets (2–3 nmol) were prepared by evaporating hexane from the purified stock solutions. A stock solution of MPA in methanol (~0.2 M, pH ~10) was prepared by dissolving MPA in methanol and adjusting pH by adding TMAOH. This MPA stock solution (0.2 mL) was added to the NC pellets with additional methanol (2 mL), and the mixture was stirred and sonicated until the NCs were homogeneously dispersed. The MPA-coated NCs were precipitated by adding ethyl acetate, and the NC pellets were rinsed and dried with acetone. Finally, the NCs were dissolved in a 0.2 mM NaOH aqueous solution for absorption spectrum measurements.

Supplementary References

[1] Chen, O. *et al.* Synthesis of metal-selenide nanocrystals using selenium dioxide as the selenium precursor. *Angew. Chem. Int. Ed.* **47**, 8638-8641 (2008).

[2] Carbone, L. *et al.* Synthesis and micrometer-scale assembly of colloidal CdSe/CdS nanorods prepared by a seeded growth approach. *Nano Lett.* **7**, 2942-2950 (2007).

[3] Qu, L. H. & Peng, X. G. Control of photoluminescence properties of CdSe nanocrystals in growth. *J. Am. Chem. Soc.* **124**, 2049-2055 (2002).

[4] Lim, S. J., Kim, W., Jung, S., Seo, J. & Shin, S. K. Anisotropic Etching of Semiconductor Nanocrystals. *Chem. Mater.* **23**, 5029-5036 (2011).

[5] Lim, S. J., Kim, W. & Shin, S. K. Surface-Dependent, Ligand-Mediated Photochemical Etching of CdSe Nanoplatelets. *J. Am. Chem. Soc.* **134**, 7576-7579 (2012).
